# Therapeutically Induced Lymphangiogenesis Is Ineffective in Resolving Established Kidney Disease in Mice

**DOI:** 10.34067/KID.0000000671

**Published:** 2024-12-17

**Authors:** Saranya Kannan, Thien T. Phan, Heidi A. Creed, Andrea J. Reyna, Gaurav Baranwal, Aubrie L. Rich, Dawson L. Weiss, Joseph M. Rutkowski

**Affiliations:** Department of Medical Physiology, Texas A&M University School of Medicine, Bryan, Texas

**Keywords:** AKI, CKD, endothelial cells, endothelium, nephrotic syndrome, VEGF

## Abstract

**Key Points:**

- CKD is a state of unresolved kidney inflammation.
- Lymphatic vessels and lymphangiogenesis regulate inflammation, and thus, more lymphatics could potentially resolve inflammation and CKD progression.
- Induction of kidney-specific lymphangiogenesis in three mouse CKD models did not improve kidney function and has the potential to worsen CKD.

**Background:**

CKD counts AKI as one of its many underlying causes. Lymphatic vessels are important in modulating inflammation postinjury. Manipulating lymphatic vessel expansion thus has the potential to alter CKD progression. Previously, we demonstrated that renal lymphatic expansion before injury reduced CKD progression after an AKI. Here, we test whether inducing lymphangiogenesis affects established CKD.

**Methods:**

After CKD progression, kidney lymphatics were expanded by transgenic induction of kidney-specific overexpression of vascular endothelial growth factor-D in aristolochic acid (AA) nephropathy and cisplatin injury aggravated with chronic high phosphate diet (CisPi) models or by infusion of kidney-targeting nanoparticles loaded with the vascular endothelial growth factor receptor-3 specific ligand vascular endothelial growth factor-C C156S in a progressive proteinuria (POD) model. Renal fibrosis and lymphatic density were determined by picrosirius red staining and immunofluorescence, respectively. Renal function was assessed by creatinine clearance rate, serum creatinine, BUN, and urinary albumin-creatinine ratio. Renal proinflammatory and fibrotic markers expression were measured by quantitative RT-PCR.

**Results:**

Kidney-specific overexpression of vascular endothelial growth factor-D+ mice demonstrated expanded renal lymphatics, while nanoparticles treatment minimally expanded lymphatics. In neither the AA nor POD model did lymphangiogenesis improve renal function or fibrosis. AA mice showed decreased *Tgfb1* expression and POD mice showed increased *Col4a1* expression. Expansion worsened function in CisPi CKD and increased fibrosis. CisPi kidneys also demonstrated increased expression of *Mcp-1*, *Il1b*, *Col1a1*, and *Tgfb1* and increased macrophage numbers.

**Conclusions:**

Therapeutically induced lymphatic expansion is ineffective in resolving established CKD and has the potential to further worsen CKD progression.

## Visual Abstract

**Figure:**
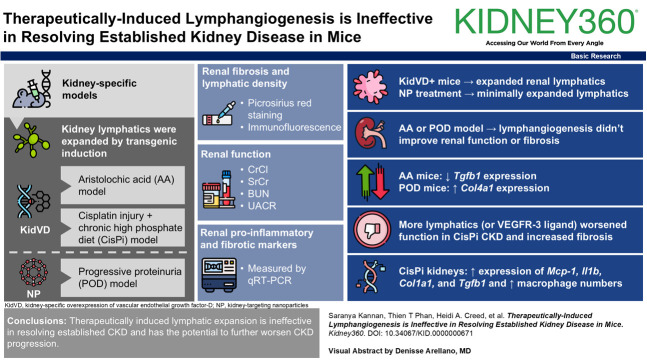

## Introduction

CKD is one of the major contributors of global mortality.^1^ CKD is characterized by progressive loss of renal function and can eventually lead to renal failure. An increasing incidence of AKI adds to the burden on the kidney, resulting in CKD. Patients with AKI are more susceptible to future CKD and may potentially progress into more advanced stages of CKD.^2–10^ An inadequate or maladaptive repair response post-AKI likely contributes to this increased risk for CKD development.^11,12^ Lymphatic vessels and lymphangiogenesis are important in the maintenance of tissue homeostasis and the resolution of inflammation postinjury.^13–15^ Increased expression of the primary lymphatic growth factors, vascular endothelial growth factor-C and -D (VEGF-C/D), and lymphangiogenesis occur naturally as part of the inflammatory response after a kidney injury and studies manipulating lymphatics have demonstrated both beneficial and detrimental effects.^16,17^ Could lymphangiogenesis be a potential therapeutic target in established kidney disease?

Lymphatic vessels in the kidney have been implicated in various kidney functions including renal development, tissue fluid regulation, and immune responses, making them a potential factor in the progression and persistence of renal disease.^18–24^ Several studies have reported an association between lymphangiogenesis and renal fibrosis development in CKD, with increasing severity of proteinuria, lower eGFR, and increased interstitial inflammation correlating both with more lymphatics and more fibrosis.^25–30^ However, some studies have demonstrated a protective effect of renal lymphangiogenesis in CKD. It has been suggested that increased vascular endothelial growth factor receptor-3 (VEGFR-3) signaling and lymphangiogenesis may improve kidney disease progression by removing the immune cell infiltrates, proinflammatory cytokines, inflammation-induced interstitial fluid accumulation, and cellular debris.^31–35^ In concordance with these studies, we previously reported that renal lymphatic expansion prior to injury provides protection from CKD progression by altering the injury recovery inflammatory and immune response.^36^ However, a dearth of knowledge remains on the therapeutic potential of renal lymphatic expansion in treating CKD.

Induction of lymphangiogenesis has largely proven to be beneficial in instances of chronic inflammation.^37^ We hypothesized that increasing renal lymphatic density could ameliorate established CKD. We tested this hypothesis in three CKD models reflecting the human CKD etiologies. The aristolochic acid (AA) causes acute tubular necrosis in the early phase, followed by progressive tubulointerstitial fibrosis, which closely mimics the pathophysiology of CKD.^38,39^ Cisplatin injury aggravated with chronic high phosphate diet (CisPi) effectively recapitulates the progression of AKI to CKD by stressing the kidney with high dietary phosphate consumption (which is to be avoided by CKD patients).^40^ We used a transgenic mouse model of kidney-specific overexpression of VEGF-D (KidVD)^36,41–43^ and induced renal lymphatic expansion after kidney disease progression in these two CKD mouse models. Given that models of rapidly progressing ESKD offer limited opportunity for effective therapeutic intervention, we used a podocyte-specific apoptosis through targeted activation of caspase-8 (POD-ATTAC) mice model of nephrotic syndrome, which allows control over the degree of injury, to check the therapeutic efficacy of a kidney targeted nanoparticle (NP) delivering a prolymphangiogenic growth factor.^44–48^ Through the various models, we identify that increased VEGFR-3 signaling and lymphangiogenesis are incapable of improving kidney function in established kidney disease in these models.^18–24^

## Methods

### Mouse Models

The KidVD mouse line (*Ksp*-rtTA×TRE-VEGFD) has been previously described.^41,43,49^ KidVD mice overexpress VEGF-D, specifically in the kidney after doxycycline administration. Mice heterozygous for both transgenes were used as KidVD mice, with littermates lacking one or both of the transgenes serving as controls. POD-ATTAC mice undergo caspase-8 mediated podocyte apoptosis after administration of a dimerizing agent and have been well characterized as a model of proteinuric kidney disease.^36,45,47,50^ All mice were either derived on a C57Bl/6 background or backcrossed 10+ generations. Male and female mice were used throughout all studies, with the average age at the time of initial injury being 10–12 weeks. Mice were provided *ad libitum* access to standard chow and drinking water throughout the study. All animal procedures were approved by the Institutional Animal Care and Use Committee at Texas A&M University.

### Injury Models

For AA nephropathy, KidVD mouse cohorts (KidVD+ and littermate controls) were intraperitoneally injected with saline or AA I (2 mg/kg bw; MilliporeSigma) once daily for five consecutive days.^38^ Doxycycline hyclate was provided in the drinking water (0.2 mg/ml; Sigma) to all mice starting at 28 days after the final AA injection. Cohorts were then maintained on doxycycline drinking water for 3 weeks.

Cisplatin injury aggravated with CisPi has been previously described as a reproducible model of CKD.^40^ KidVD cohorts (KidVD+ and littermate controls) received a single intraperitoneal saline or cisplatin (10 mg/kg bw) injection (Accord Healthcare) and were placed on standard chow or a 2% inorganic phosphate diet 2 weeks postinjury (Teklad TD.220660; 42 g of calcium phosphate and 14 g of potassium phosphate per kilogram diet) for 13 weeks to aggravate injury, and all mice received doxycycline hyclate (0.2 mg/ml) in their drinking water, along with phosphate diet for the last 3 weeks of experimental period.

Podocyte apoptosis was induced in POD-ATTAC mice by a single intraperitoneal injection of the dimerizering agent AP20187 (0.4 *µ*g/g bw), prepared according to the manufacturer’s instruction (Takara Bio, Inc.). Starting 28 days later, mice received a 100 *µ*l tail vein infusion containing micellar NPs loaded with 500±17 *µ*g of the VEGFR-3-specific mutant of VEGF-C (VEGF-C c156s; R&D Systems) twice a week for 3 weeks. The NP formulation and kidney-specific targeting of these particles has been previously described.^51^

Both the littermate controls and KidVD+ animals received doxycycline to control for any off the target effects, and POD mice receiving dimerizer were used as controls in POD-ATTAC studies.

### Urine Collection and Analysis

Mice were placed into individual diuresis metabolic chambers (Hatteras Instruments) and allowed to acclimate overnight. After acclimatization, urine was collected over a 24-hour period (concluding with euthanasia) and 24-hour urine volumes were measured. Total urine protein concentrations were determined by bicinchoninic acid assay (Thermo Scientific), with urine diluted 1:20–1:200, as necessary in PBS. Specific urine albumin concentrations were quantified using an Albuwell M kit (Exocell), with sample dilutions of 1:20–1:200 in assay buffer. Creatinine analysis was conducted by the UAB-UCSD O’Brien Center for AKI Research using liquid chromatography-tandem mass spectrometry.

### Serum Analysis

Serum creatinine (SrCr) values were measured as in urine and used to calculate the creatinine clearance. BUN was measured using the Infinity Urea Liquid Stable Reagent (Thermo Fisher Scientific; TR12421) according to the manufacturer’s instruction.

### Renal Lymphatic Quantification

Formalin-fixed, paraffin-embedded kidney sections (5–7 *µ*m thick) were deparaffinized, rehydrated, and permeabilized with 0.1% TritonX solution. Sections were blocked with Aquablock (East Coast Bio) before being immunolabelled with lymphatic endothelial cell marker lymphatic vessel endothelial hyaluronan receptor 1 (LYVE-1; R&D Systems, AF2126). Samples were incubated with Alexafluor 488 conjugated secondary antibody (Invitrogen) for an hour at room temperature. Slides were mounted with ProLong Gold antifade reagent containing 4′,6-diamidino-2-phenylindole (Invitrogen). An Olympus BX51 fluorescence microscope with an Olympus Q5 camera was used for imaging, and representative images were captured at 10× magnification using Olympus CellSens imaging software (Olympus). Two independent blind investigators counted the number of LYVE-1+ lumen surrounding the interlobular arteries at the cortex and corticomedullary junctions. Arteries were identified as structures with a clear lumen surrounded by a prominent muscular layer (tunica media), seen both in background color and 4′,6-diamidino-2-phenylindole nuclear alignment of muscle cells. Owing to the density of lymphatic vessels in KidVD mice, four nonoverlapping images were captured from within the renal cortex at 10× magnification and ImageJ software (version 1.53k; National Institutes of Health) was used to determine the area value measurements to measure the total number of LYVE-1+ pixels after setting the threshold for positive labeling. To measure lymphatic vessel size, images of all artery-associated lymphatic vessels within the cortex were collected from each kidney sections at 20×. Lymphatic vessels were identified by the presence of LYVE-1 staining. ImageJ was used to collect the length in pixels of the widest point of all lumen-containing lymphatic vessels.

### Quantification of Macrophage Numbers

Macrophages were identified by labeling for F4/80 (Abcam, AB300421) with secondary detection. Macrophages were identified as F4/80+ cells with a roughly circular or ovoid morphology. Representative images were captured at 10× and 20× magnification using Olympus CellSens imaging software. Investigators blinded to the experimental conditions counted the number of F4/80+ macrophages in the whole section under 20× magnification.

### Quantification of Podocyte Numbers

Podocytes were identified by labeling with antibodies against podoplanin (R&D Systems, AF3244) and Wilms tumor-1 (WT-1; Santa Cruz Biotechnology, SC-7385) after citrate buffer antigen retrieval. Alexafluor 488 or 594 (Invitrogen) secondary antibodies were used to visualize the podocytes. Detection, mounting, and imaging were performed as described above. An investigator blinded to the experimental condition counted the number of WT-1-stained nucleus surrounded by podoplanin, as described previously.^45^ In the instance of injured glomeruli with reduced podoplanin expression, glomerular WT-1+ nuclei were considered as podocytes. Total WT-1+ cell counts were then divided by the average glomerular cross-sectional area on the kidney section measured in ImageJ software. The final podocyte counts are therefore reported as WT-1+ cells/glomerular area (*µ*m^2^) ×10^−5^.

### Interstitial Fibrosis Determination

Picrosirius (PS) red staining was performed on renal tissue sections at Saffron Scientific Histology Services, LLC (Carbondale, IL). Representative brightfield cortex images were captured at 10× magnification avoiding tissue edges, medulla, and the renal hilus. ImageJ software was used to quantify the collagen-dense positive area for a range of red hues identified on a positive fibrous streak from a given staining batch. Total positive pixels are presented as a percentage total image area and averaged per mouse.

### Quantitative RT-PCR

Total RNA was isolated from mouse kidneys using Zymo Direct-zol RNA Miniprep Plus kit as per the manufacturer’s instructions (Zymo Research). cDNA synthesis using 1 *µ*g total RNA was performed using the iScript cDNA Synthesis kit instructions (Bio-Rad Laboratories, Inc.,). Five microliters quantitative PCR reactions were performed using iTaq Universal SYBR Green Supermix (Bio-Rad) and primers (5 *μ*M; Sigma-Aldrich), along with kidney cDNA in duplicates on a QuantStudio6 Flex Real-Time PCR system (Applied Biosystems). Fold changes compared with control samples (defined in each Figure Legend) were calculated using the 2^*ΔΔ*CT^ method with *Ubc* as an endogenous control. Forward and reverse primer sequences are listed in Table 1.

**Table 1:** Primer sequences for quantitative RT-PCR analysis of murine renal tissue

Target	Forward (5′ to 3′)	Reverse (5′ to 3′)
*Ubc*	GCC​CAG​TGT​TAC​CAC​CAA​GAA​G	GCT​CTT​TTT​AGA​TAC​TGT​GGT​GAG​GAA
*Lyve1*	CTG​ACA​AGC​AGT​TTC​AGG​CTT​GGT	TTC​AGC​CCA​CAC​TCC​GCT​ATA​CAT
*Pdpn*	AGA​GAA​CAC​GAG​AGT​ACA​ACC	CAA​CAA​TGA​AGA​TCC​CTC​CGA​C
*Prox1*	AGA​AGG​GTT​GAC​ATT​GGA​GTG​A	TGC​GTG​TTG​CAC​CAC​AGA​ATA
*Ccl21*	CCC​TGC​TTC​AAC​AAT​TAC​ATC​T	CCT​GCT​GTC​TCC​TTC​CTC​ATT​CC
*Nrp2*	GCT​GGC​TAC​ATC​ACT​TCC​CC	CAA​TCC​ACT​CAC​AGT​TCT​GGT​G
*Vegfr3*	ATC​AGA​AGA​TCG​GGC​GCT​GTT​GTA	TGT​GTC​ATG​TCC​GCC​CTT​CAG​TTA
*Vegfc*	CAG​TGT​CAG​GCA​GCT​AAC​AAG	GGT​CCA​CAG​ACA​TCA​TGG​AA
*Vegfd*	AAA​TCG​CGC​ACT​CTG​AGG​A	TGG​CAA​GAC​TTT​TGA​GCT​TCA​A
*Tnfa*	GAG​AAA​GTC​AAC​CTC​CTC​TCT​G	GAA​GAC​TCC​TCC​CAG​GTA​TAT​G
*Mcp1*	ACT​CAC​CTG​CTG​CTA​CTC​AT	CTA​CAG​CTT​CTT​TGG​GAC​A
*Il6*	CCA​AGA​GGT​GAG​TGC​TTC​CC	CTG​TTG​TTC​AGA​CTC​TCT​CCC​T
*Il1b*	ATG​ATG​GCT​TAT​TAC​AGT​GGC​AA	GTC​GGA​GAT​TCG​TAG​CTG​GA
*Ngal*	CTC​AGA​ACT​TGA​TCC​CTG​CC	TCC​TTG​AGG​CCC​AGA​GAC​TT
*Fn*	ACA​AGG​TTC​GGG​AAG​AGG​TT	CCG​TGT​AAG​GGT​CAA​AGC​AT
*Col1a1*	GCC​AAG​AAG​ACA​TCC​CTG​AA	GTT​TCC​ACG​TCT​CAC​CAT​TG
*Col4a1*	GAC​AGC​CAG​GTT​TGA​CAG​GT	GGC​AGC​TCT​CTC​CTT​TCT​GA
*aKlotho*	ACA​AAG​AAG​TGG​GCC​GAG​AGA	CGG​TGA​AAT​AGG​GCA​AAA​GA
*Acta2*	GAC​GCT​GAA​GTA​TCC​GAT​AGA​ACA​C	CCA​CCA​TCT​CCA​GAG​TCC​AGC​ACA​AT
*Tgfb1*	CAG​CAC​GGC​CCC​AAT​GTA​T	GGG​ACC​TTT​TCA​TAT​CCA​GGA​CA
*F480*	CTG​TAA​CCG​GAT​GGC​AAA​CT	CTG​TAC​CCA​CAT​GGC​TGA​TG
*Cd68*	TGT​CTG​ATC​TTG​CTA​GGA​CCG	GAG​AGT​AAC​GGC​CTT​TTT​GTT​A

Target indicates RNA of interest using the common gene name.

### Statistical Analysis

Statistical analysis was performed using GraphPad Prism, version 8.0.1. Differences between two groups were assessed by an unpaired *t* test and between three or more groups with a one-way ANOVA, followed by Tukey multiple comparisons test. The statistical test performed and final number of samples per group are stated in the figure legends. All data are displayed as the mean±SD. A *P* < 0.05 was considered significant.

## Results

### Impact of Different Kidney Disease Models on Renal Lymphangiogenesis

**Figure 1:**
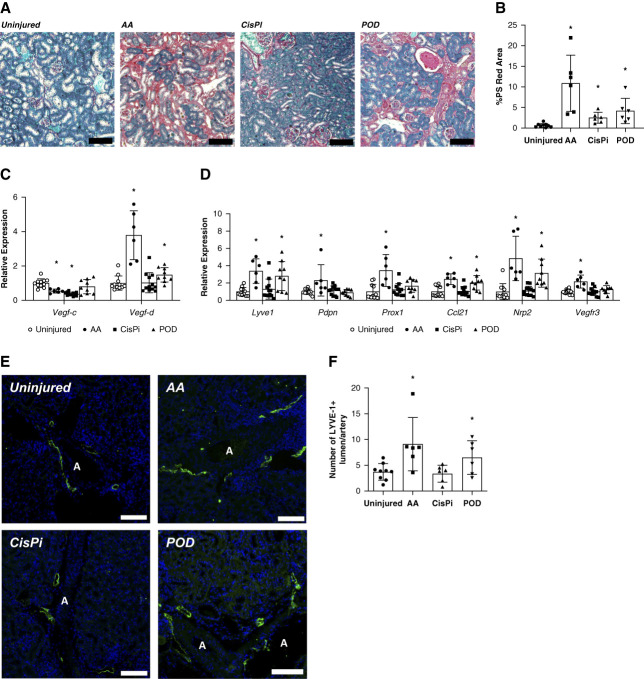
**Renal lymphangiogenesis after CKD.** (A) PS red staining kidney sections of mice. Scale bars=100 *µ*m. (B) Percentage of cortex images positive for intense PS red staining. *n*=9 uninjured, six AA, six CisPi, six POD. (C) mRNA expression levels of lymphangiogenic growth factors in the kidney at day 60 relative to uninjured. *n*=11 uninjured, six AA, 12 CisPi and nine Podo. (D) mRNA expression levels of lymphatic endothelial markers in the kidney at day 60 relative to uninjured. *n*=11 uninjured, six AA, 12 CisPi and nine POD. (E) Immunofluorescence of renal cortical lymphatics labeled for LYVE-1 (green) and DAPI (blue). Scale bars=100 *µ*m. (F) Count of LYVE‐1+lumen/artery on tissue sections of the renal cortex (A indicates an artery). *n*=9 uninjured, six AA, six CisPi, six POD. All data are represented as ±SD. Statistical comparisons were made using unpaired *t* test. * *P* < 0.05 versus uninjured mice. AA, aristolochic acid; CisPi, chronic high phosphate diet; DAPI, 4′,6-diamidino-2-phenylindole; LYVE-1, lymphatic vessel endothelial hyaluronan receptor 1; POD, proteinuria; PS, picrosirius; VEGF-C, vascular endothelial growth factor-C.

Several studies have demonstrated a positive association between renal fibrosis and lymphangiogenesis during CKD progression.^25,26,52^ The extent of renal fibrosis in the three injury models after disease progression was evaluated by PS red staining, and a significant increase in positive staining was observed in all three models compared with uninjured kidneys demonstrating fibrotic CKD progression (Figure 1, A and B). All three models demonstrated significantly higher fibrotic area compared with the uninjured (Figure 1B). Gene expression levels for the lymphangiogenic growth factors VEGF-C and VEGF-D were not universally elevated, despite the fibrosis, with only VEGF-D being significantly elevated in the AA and POD kidneys (Figure 1C). Expression of lymphatic endothelial markers *Lyve1*, *Pdpn Prox1*, *Ccl21*, *Vegfr3*, and *Nrp2* were to varying degrees significantly elevated in AA and POD kidneys, but surprisingly unchanged in CisPi kidneys (Figure 1D). To determine whether CKD induced renal lymphangiogenesis, LYVE-1 immunolabelling was performed and quantified. Cortical lymphatics are largely found near the interlobular arteries in a normal kidney.^53^ Consistent with the significant fibrosis and lymphatic gene expression, the AA and POD model showed a significant increase in LYVE-1+ lumen count, while CisPi kidneys demonstrated no change in lymphatic vessel number (Figure 1, E and F). These models therefore provide a range of etiologies and structural differences in which to test inducing lymphangiogenesis.

### Inducing Kidney Lymphangiogenesis Does Not Affect AA-Induced Renal Dysfunction

**Figure 2:**
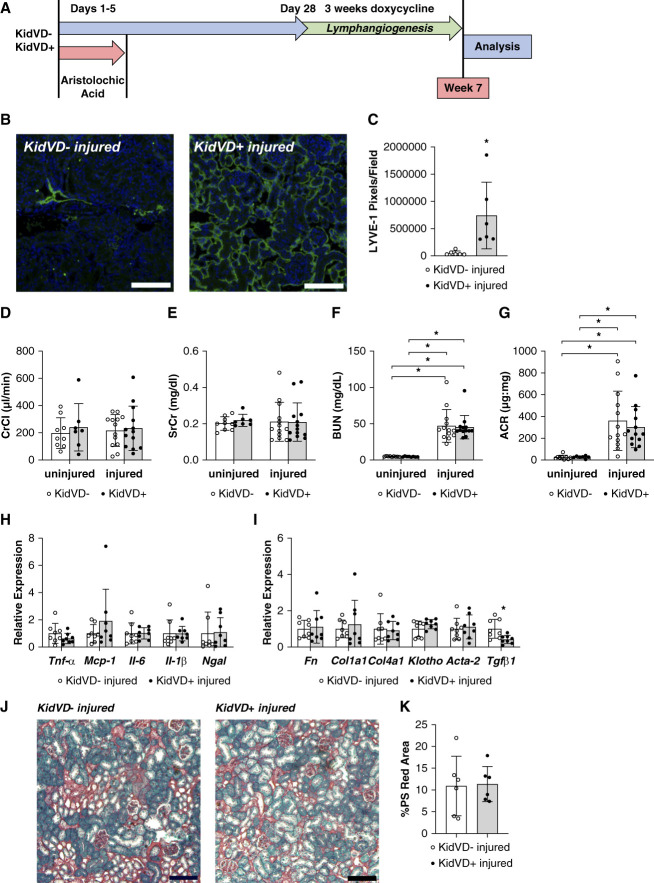
**Effect of expanded lymphatics on AA-induced CKD outcomes.** (A) Timeline showing the injury and treatment protocol. (B) Immunofluorescence of renal cortical lymphatics labeled for LYVE-1 (green) and DAPI (blue). Scale bars=100 *µ*m. (C) Average LYVE-1+ pixels on four different fields/section. *n*=6 KidVD− injured, six KidVD+ injured. (D) CrCl. (E) SrCr. (F) BUN. (G) Urinary ACR *n*=9 KidVD− uninjured, seven KidVD+ uninjured 13 KidVD− injured, 13 KidVD+ injured. (H) mRNA expression levels of inflammatory markers in the kidney at termination relative to control littermates. *n*=8 KidVD− injured, eight KidVD+ injured. (I) mRNA expression levels of fibrosis markers in the kidney at termination relative to control littermates. *n*=8 KidVD− injured, eight KidVD+ injured. (J) PS red staining kidney sections of mice. Scale bars=100 *µ*m. (K) Percentage of cortex images positive for intense PS red staining. *n*=6 KidVD− injured, six KidVD+ injured. All data are represented as ±SD. Statistical comparisons were made using unpaired *t* test or one-way ANOVA. * *P* < 0.05. ACR, albumin-creatinine ratio; CrCl, creatine clearance; KidVD, kidney-specific overexpression of vascular endothelial growth factor-D; MCP-1, monocyte chemoattractant protein-1; SrCr, serum creatinine.

Previously, we demonstrated that increased lymphatic density before injury affords protection from CKD progression after AKI.^36^ AAs induce the development of CKD and have been used as a model to study the AKI to CKD transition.^38^ To test whether inducing renal lymphatic expansion could help to resolve established kidney disease, renal lymphangiogenesis was genetically induced for 3 weeks after disease progression in AA nephropathy-induced KidVD mice (Figure 2A). KidVD+ mice demonstrated significant kidney lymphatic expansion as visualized by LYVE-1+ lymphatic vessel density imaging and quantitation compared with their injured KidVD-littermates (Figure 2, B and C). After 3 weeks of induced lymphangiogenesis (approximately 7 weeks postinjury), renal function measured by creatinine clearance (Figure 2D) and SrCr (Figure 2E) remained within the uninjured range after disease progression with no significant change after lymphatic expansion. However, the AA mice demonstrated renal dysfunction with elevated BUN (Figure 2F) and urinary albumin-creatinine ratio (ACR; Figure 2G), but no significant difference was measured between the injured KidVD+ mice and their injured KidVD-littermates. Kidney inflammation was assessed by measuring cortical RNA expression for the cytokines TNF*α*, monocyte chemoattractant protein-1 (MCP-1), IL-6, IL-1b, and Ngal, with no significant differences identified between KidVD− and KidVD+ kidneys after disease progression (Figure 2H). Similarly, fibrosis gene expression was largely unchanged with no differences measured in *Fn*, *Col1a1*, *Col4a1*, *Klotho*, or *Acta2*; however, *TgfB1* was significantly decreased in KidVD+ kidneys after disease progression (Figure 2I). Further quantitation of PS red area confirmed no change in fibrosis with induced lymphangiogenesis in injured KidVD+ mice (Figure 2, J and K). Overall, these data demonstrate that genetically expanding renal lymphatics does not positively affect kidney disease progression in AA CKD.

### Expanding Lymphatics Worsens the CisPi Model Induced CKD

**Figure 3:**
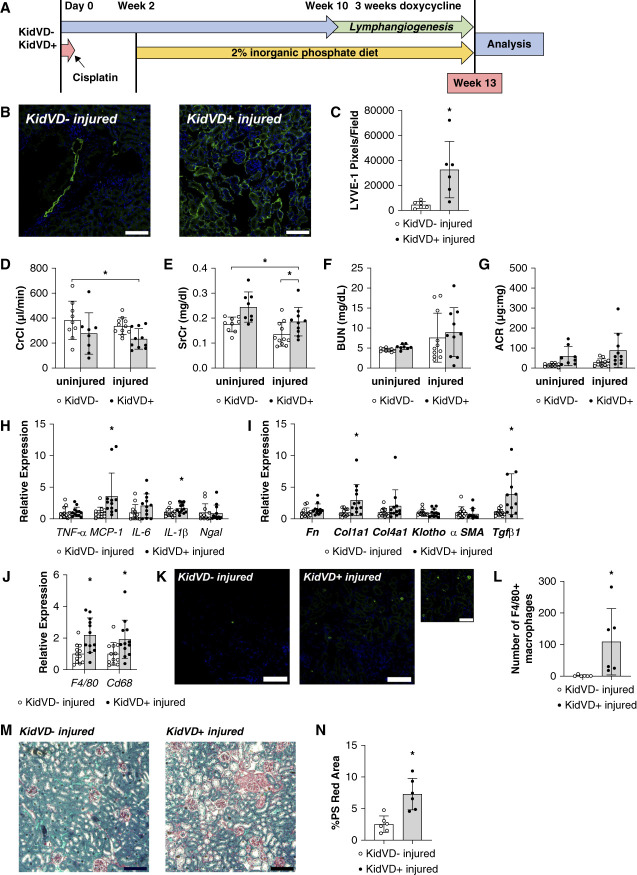
**Effect of expanded lymphatics on chronic phosphate and cisplatin-induced CKD outcomes.** (A) Timeline showing the injury and treatment protocol. (B) Immunofluorescence of renal cortical lymphatics labeled for LYVE-1 (green) and DAPI (blue). Scale bars=100 *µ*m. (C) Average LYVE-1+ pixels on four different fields/section. *n*=6 KidVD− injured, six6 KidVD+ injured. (D) CrCl. (E) SrCr. (F) BUN. (G) Urinary ACR *n*=9 KidVD− uninjured, eight KidVD+ uninjured 11 KidVD− injured, ten KidVD+ injured. (H) mRNA expression levels of inflammatory markers in the kidney at termination relative to control littermates. *n*=12 KidVD− injured, 12 KidVD+ injured. (I) mRNA expression levels of fibrosis markers in the kidney at termination relative to control littermates. *n*=12 KidVD− injured, 12 KidVD+ injured. (J) mRNA expression levels of macrophage markers in the kidney at termination relative to control littermates. *n*=12 KidVD− injured, 12 KidVD+ injured. (K) Immunofluorescence of renal macrophages labeled for F4/80 (green), and DAPI (blue). Scale bars=100 *µ*m; 50 *µ*m for 20× inset (these images represent specific fields within the kidney sections and may not fully reflect the heterogeneous distribution of macrophages observed across entire sections. Quantification data are based on counts from whole kidney sections). (L) Count of F4/80+ macrophages per section after injury *n*=6 KidVD− injured, six KidVD+ injured. (M) PS red staining kidney sections of mice. Scale bars=100 *µ*m. (N) Percentage of cortex images positive for intense PS red staining. *n*=6 KidVD− injured, 6 KidVD+ injured. All data are represented as ±SD. Statistical comparisons were made using unpaired *t* test or one-way ANOVA. * *P* < 0.05.

Because the underlying etiology and degree of injury in each disease model varies, we next tested the effect of genetically induced renal lymphangiogenesis after disease progression in the CisPi model of CKD development (Figure 3A). Cisplatin injection coupled with high dietary phosphate induced CKD disturbs mineral metabolism mimicking human CKD.^40^ The expected increase in renal lymphatic density was verified by LYVE-1 immunolabelling and was significantly higher in the kidneys of KidVD+ mice compared with their injured KidVD− littermates after disease progression (Figure 3, B and C). Surprisingly, induction of VEGF-D overexpression for 3 weeks significantly decreased the creatinine clearance (Figure 3D) and increased the SrCr concentration (Figure 3E), indicating decreased renal function with induced lymphangiogenesis after disease progression. BUN and ACR measurements, however, were not worsened in KidVD+ mice after the disease progression (Figure 3, F and G). Inflammatory marker gene expression for TNF*α*, IL-6, and Ngal remained unchanged, although gene expression for MCP-1 and IL-1b were significantly increased in the cortex of KidVD+ kidneys compared with their littermate controls after disease progression (Figure 3H). Expression of *Col1a1* and *Tgfβ1* were significantly increased in KidVD+ kidneys compared with their injured KidVD-littermates, suggesting an increase in fibrosis (Figure 3I). With elevated MCP-1 and fibrosis, we further investigated the presence of macrophages in the heightened inflammation by measuring RNA expression for F4/80 and Cd68 and identified a significant increase in the KidVD+ kidneys after disease progression (Figure 3J). The increase in macrophage number was further verified by F4/80 immunolabelling and was significantly higher in the kidneys of injured KidVD+ mice (Figure 3, K and L). Concordant with these findings, quantitation of PS red area demonstrated increased fibrosis in KidVD+ mice (Figure 3, M and N). Expanded lymphatics through overexpression of VEGF-D after kidney disease initiation thus seems to aggravate the kidney and worsen CKD outcomes in the CisPi model.

### Kidney VEGF-C Deliver Does Not Alter Renal Function in Established Nephrotic Syndrome

**Figure 4:**
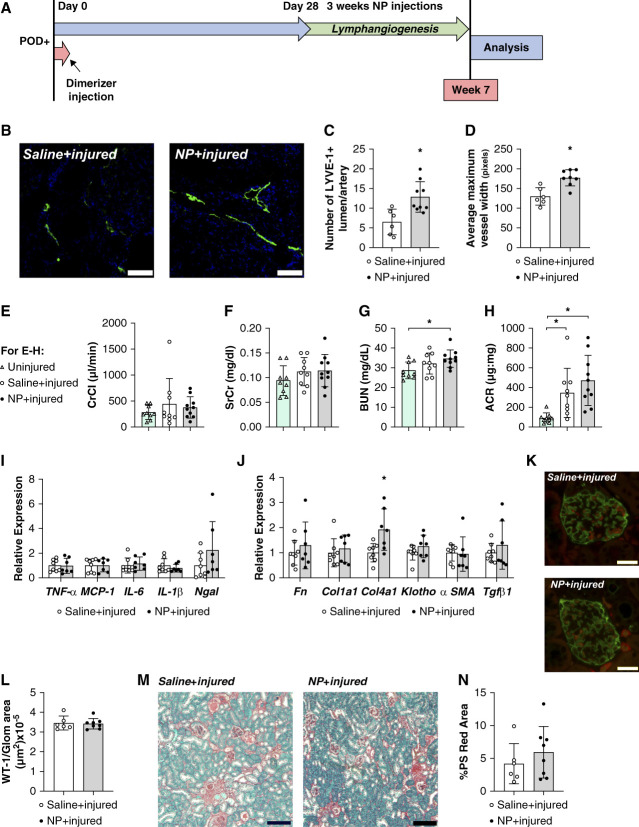
**Effect of treatment with VEGF-C in NPs on selective podocyte injury-induced CKD outcomes.** (A) Timeline showing the injury and treatment protocol. (B) Immunofluorescence of renal cortical lymphatics labeled for LYVE-1 (green), and DAPI (blue). (C) Count of LYVE‐1+lumen/artery on tissue sections of the renal cortex. *n*=6 saline+injured, eight NPs+injured. (D) Maximal renal lymphatic vessel width of lumen-containing lymphatic vessels within the cortex. *n*=6 saline+injured, eight NPs+injured. (E) CrCl. (F) SrCr. (G) BUN. (H) Urinary ACR *n*=9 uninjured nine Saline+injured, ten NPs+uninjured. (I) mRNA expression levels of inflammatory markers in the kidney at termination relative to saline-treated controls. *n*=8 saline+injured, seven NPs+uninjured. (J) mRNA expression levels of fibrosis markers in the kidney at termination relative to saline-treated controls. Eight saline+injured, seven NPs+uninjured. (K) Immunofluorescence imaging of WT‐1 for podocyte nuclei (red), podoplanin (green), and DAPI (blue) at 60 days postinjury. Scale bars=20 *µ*m. (L) Count of WT-1+ podocyte nuclei per glomerulus (WT‐1/glomerular area ×10^−5^) following injury. *n*=6 saline+injured, eight NPs+injured. (M) PS red staining kidney sections of mice. Scale bars=100 *µ*m. (N) Percentage of cortex images positive for intense PS red staining. *n*=6 saline+injured, eight NPs+injured. All data are represented as ±SD. Statistical comparisons were made using unpaired *t* test or one-way ANOVA. * *P* < 0.05. NP, nanoparticle; VEGF-C, vascular endothelial growth factor-C; WT‐1, Wilms tumor-1.

To test the effect of augmenting lymphatic expansion on established kidney disease using a potentially translatable approach, we used a kidney-specific NP to deliver the VEGFR-3 specific mutant of VEGF-C (VEGF-C c156s) in the POD-ATTAC mouse model. The C156S mutation in VEGF-C makes it specific to VEGFR-3 and is the standard in the lymphatic field for targeting VEGFR-3 activation. POD-ATTAC mice have been previously characterized as a model of proteinuric nephrotic syndrome with many similarities to human disease progression.^44,46,54,55^ One month after podocyte injury, the previously described micellar NPs^51^ were delivered for 3 weeks (Figure 4A). NP delivery of VEGF-C c156s increased the LYVE-1 positive lumen count/artery and also increased the maximal lymphatic vessel width, demonstrating lymphatic vessel hyperplasia in these mice (Figure 4, B–D). There was no significant change in the creatinine clearance and SrCr values after the NP treatment (Figure 4, E and F). NP treatment after injury did result in significantly increased BUN levels (Figure 4G). POD-ATTAC mice demonstrate increased ACR values; however, this therapeutic approach did not result in any change (Figure 4H). Similarly, there were no significant changes in the gene expression of inflammatory markers TNF*α*, MCP-1, IL-6, IL-1b, and Ngal. Similarly, no changes in the fibrosis-associated genes *Fn*, *Col1a1*, *Klotho*, *Acta2*, or *TgfB1* were measured (Figure 4, I and J). NP delivery did cause a significant increase in *Col4a1* expression. Further quantitation of PS red area confirmed no change marked changes in fibrosis after NP treatment (Figure 4, M and N). Previous studies have demonstrated a restoration of podocytes with improved injury response in the POD-ATTAC model; therefore, podocyte numbers were quantified.^36,56^ No significant changes in the podocyte density were measured after NP treatment (Figure 4, K and L). Taken together, these data suggest that NP delivery of a VEGFR-3 ligand affects lymphatic vessel growth, but has little effect on the kidney function.

## Discussion

The substantial increase in global CKD burden and the unavailability of a cure motivates the need for new approaches. We previously demonstrated that expanding renal lymphatics before injury affords protection from CKD progression after AKI.^36^ We have also demonstrated that induction of kidney lymphangiogenesis, both genetically and using NP delivery of a VEGFR-3 ligand, can reduce established hypertension and alter the immune cell populations in the kidney.^36,51^ In this study, we sought to test whether expanded renal lymphatics after the onset of kidney disease could improve kidney function or inflammation. Using multiple models and approaches, our data demonstrate that expanding renal lymphatics is ineffective toward improving the kidney.

Renal fibrosis is the predominant pathophysiologic change observed with the progression of CKD and varies across different stages and disease models.^57,58^ In this study, both AA and POD models showed a significant increase in collagen deposition depicting renal fibrosis and progressive CKD in concordant with previous studies.^45,59–61^ In line with the varying degree of renal fibrotic progression, differential effect on lymphatic expansion was observed among all three models. CKD progression is accompanied with a parallel increase in renal lymphatic vessel density,^25–27^ through increased production of lymphangiogenic ligand VEGF-D, which is often implicated in inflammation, in AA and POD mice.^62–65^ A review of several studies highlights a positive correlation between fibrosis and lymphangiogenesis,^66^ with one study demonstrating that renal tubular activated fibroblast preferentially increase lymphatic endothelial cell proliferation over the blood endothelial cells.^67^ Whether fibrosis drives lymphangiogenesis or *vice versa* is unclear.

While studies inducing lymphangiogenesis to ameliorate skin inflammation have been nearly always positive, studies manipulating lymphatic vessels and lymphangiogenesis after renal injury have yielded mixed results. In some studies, enhanced renal lymphatic proliferation has been reported to be beneficial by alleviating fibrosis through removal of inflammatory mediators and accelerates the resolution of inflammation.^32–34^ In one study, expanded lymphatics in a polycystic kidney disease model reduced the cystic disease by reducing the number of pericystic inflammatory cells.^68^ In another, podocyte overexpression of VEGF-C was able to reduce POD in diabetic nephropathy mice.^35^ Other studies, however, have reported detrimental effects of lymphangiogenesis or VEGFR-3 signaling on CKD progression by promoting intrarenal inflammation and fibrosis through an elevated proinflammatory immune response.^27,62^ VEGFR-3 ligands stimulate macrophage chemotaxis and its signaling in macrophages is detrimental in renal fibrosis.^69^ In this study, providing additional VEGFR-3 ligands or inducing lymphangiogenesis had little to no effect on kidney inflammation or fibrosis in the AA and POD models. This may be because the fibro-inflammatory tissue environment in these models was already quite elevated. In the CisPi model, in which basal fibrosis and lymphangiogenesis was insignificant, inducing VEGF-D overexpression further increased renal fibrosis and decreased renal function in the CisPi model. MCP-1 and IL-1*β* are implicated in the development and progression of renal fibrosis.^70,71^ Similarly, a positive feedback loop involving Tgf-*β*1 and collagen-1 promotes fibroblast activation.^72^ Increased MCP-1 expression in kidneys correlates with greater macrophages infiltration during kidney disease.^73^ Similarly, VEGFR-3 activation has been implicated to increase macrophage infiltration during tubulointerstitial fibrosis; it can also mediate lymphangiogenesis through M1 macrophage polarization in renal fibrosis.^69^ Increased expression of these factors, along with a huge number of macrophages in the KidVD+ CisPi mice, may have resulted in further progression of renal fibrosis and subsequent renal dysfunction. In total, our data support the previous studies implicating and correlating kidney lymphangiogenesis, fibrosis, and disease progression. Collectively, this would suggest that inhibiting VEGFR-3 in established CKD is a more positive intervention approach.

It is important to note the current findings may be limited by the specific models employed. Each injury model exhibited varying degrees of fibrosis, lymphatic growth factor upregulation, and lymphangiogenesis at baseline that influenced the effect of lymphatic expansion interventions. C57/Bl6 mice were used, which respond differently to kidney injury than other strains. The dosing of the initial injury-causing agonists was not tested, nor the amount of VEGFR-3 ligand that was overexpressed or delivered by the NP. The timing of intervention relative to disease progression, the duration and rate of lymphangiogenesis, and the CKD follow-up time were also not examined and may yield different functional and inflammatory outcomes. Specific immune populations in the kidneys or renal lymph nodes were also not examined as in past studies.

Lymphatic vessels and lymphangiogenesis pose an interesting target in chronic inflammatory diseases because of their ability to alter the tissue immune and inflammatory environment. While other models, dosing, or timings may yield a more positive outcome, from this study, we must conclude that therapeutically induced lymphangiogenesis is ineffective in resolving established CKD and has the potential to further worsen CKD progression.

## Data Availability

Partial restrictions to the data and/or materials apply. Any data will be made available upon request to the corresponding author.
